# Citations of microRNA Biomarker Articles That Were Retracted: A Systematic Review

**DOI:** 10.1001/jamanetworkopen.2024.3173

**Published:** 2024-03-21

**Authors:** Hongmei Zhu, Yongliang Jia, Siu-wai Leung

**Affiliations:** 1 Center of Gastrointestinal and Minimally Invasive Surgery, Department of General Surgery, Third People’s Hospital of Chengdu, Chengdu, Sichuan, China; 2 Medical Research Center, Third People’s Hospital of Chengdu, Affiliated Hospital of Southwest Jiaotong University, Chengdu, Sichuan, China; 3 Henan Institute of Medical and Pharmaceutical Sciences, Zhengzhou University, Zhengzhou, Henan, China; 4 Edinburgh Bayes Centre for AI Research in Shenzhen, College of Science and Engineering, University of Edinburgh, Scotland, United Kingdom

## Abstract

**Importance:**

Retraction is a tool that journals can use to deter research misconduct and alert their audience to erroneous content published in the journals. However, retracted articles may continue to damage science if they are still cited as legitimate articles.

**Objective:**

To characterize patterns of postretraction citations, particularly in microRNA biomarker research, a field with one of the highest rates of retraction.

**Evidence Review:**

Retracted scientific articles on microRNAs were retrieved from PubMed, Web of Science, and Retraction Watch between database inception and July 17, 2021, according to preestablished search strategies. Control articles with characteristics in common with retracted articles (ie, published in the same journals in the same years and months and with the same number of authors) were matched and retrieved from PubMed. Citation metrics of retractions and control articles were collected from Web of Science. PubPeer was referenced to examine the public response or comments on included retractions. Data were analyzed from September 2021 through March 2023.

**Findings:**

A total of 10 461 articles were analyzed, with 887 retractions and 9574 articles as controls. Among retracted articles, which were published from 1999 to 2021, there were 756 articles (85.23%) written by researchers affiliated with Chinese institutions. Retracted articles were cited 6327 times after retraction. Of 792 retracted articles that were cited, 621 articles (78.41%) were cited at least once after retraction and 238 articles (30.05%) were cited more often after retraction than before retraction. Overall citations (comprising citations before and after retraction) and postretraction citations accumulated over time (eg, the median [IQR] number of postretraction citations was 1 [1-2] and 23 [9-44] citations at the first 6 and 66 months, respectively, between retraction and citation retrieval). A random sample of 87 retracted articles (9.81%) recorded 478 citations after retraction, with 208 citations (43.51%) in articles published 12 months or longer after retraction. Of these citing articles, 19 articles (3.97%) mentioned the retractions. Compared with the control group of 1620 nonretracted articles, no significant differences were found in overall number of citations or citations after retraction. Among 478 articles citing retracted articles, 414 articles were found on PubMed and had matched control articles; these articles had higher odds of being subsequently retracted than 7954 matched control articles (odds ratio, 6.57; 95% CI, 3.39-12.72).

**Conclusions and Relevance:**

In this study, retraction was not associated with a reduction in citations of retracted articles, but articles that cited retracted publications had higher odds of later retraction. These findings suggest that journals may need to implement mechanisms for detection of postretraction citations.

## Key Points

**Question:**

Are retractions of articles reporting microRNA biomarkers associated with reductions in further citations?

**Findings:**

This systematic review of 887 retracted articles reporting microRNA biomarkers and 9574 control articles found that retraction was not associated with reductions in further citations and that compared with controls, publications citing retracted articles were more often retracted.

**Meaning:**

These findings suggest that better identification of postretraction citations should be implemented.

## Teaser

This systematic review investigates the association of retractions with patterns of further citations among articles reporting microRNA studies.

## Introduction

Retraction is a means that academic publishers can use to deter research misconduct and alert audience of erroneous content published in the journals.^1^ Retraction Watch recorded that the number of retracted articles increased from 41 articles in 2000 to more than 6000 articles in 2022.^2^ *Nature* reported that the number of retractions increased each year and passed 10 000 articles in 2023.^3^ The increasing prevalence of documented retractions outpaced the growth rate of publications.^3^ The situation may be more severe given that that some researchers believe that documented retractions are just the tip of the iceberg.^4^ Common reasons given for retractions included honest scientific mistakes (eg, inadvertent errors),^1^ research misconduct (eg, plagiarism, fabrication, or falsification),^5^ and various administrative errors.^6^ However, involvement of so-called paper mills, organizations selling fake work and authorships to researchers, was a major factor associated with the rapid increase of retractions in recent years.^7,8,9,10,11^

The International Committee of Medical Journal Editors (ICMJE) recommends PubMed as the verified bibliographic source for references to minimize citation errors and an authoritative source of information about retractions for articles indexed in MEDLINE.^12^ A PubMed search found that more than 6000 articles were retracted between 2020 and 2022 and that cancer, RNA, and microRNA were the most frequently appearing words in retractions after excluding words like retraction (or retracted), note, cell, pathway, human, patient, inhibits, targeting, and promotes. In the Retraction Watch database, 232 of 484 retractions (47.93%) were about microRNAs in genetics in 2020, and this proportion increased to 699 of 1026 retractions (68.13%) in 2022.^2^ These outcomes suggest that microRNA research may be problematic as a source of retractions.

Articles in 2020^13^ and 2021^14^ found that retracted articles were often cited as if they had never been retracted, although the ICMJE recommended that authors are responsible for checking that none of their references cite retracted articles except in the context of referring to the retraction.^12^ Not many journals check references in manuscripts for postretraction citations. Only recently, Wiley implemented a reference checking system to access the Retraction Watch database.^15^

To our knowledge, few articles have covered postretraction citations to evaluate the effectiveness of retractions in reductions of further citations of retracted articles. This study aimed to fill this research gap by conducting a systematic review to identify characteristics of retractions in microRNA biomarker research as a common source of recent retractions. Specifically, we examined trends of postretraction citations by comparing retracted microRNA biomarker articles with a control group of nonretracted articles and investigated whether retraction was associated with reductions in postretraction citations.

## Methods

This systematic review was registered in the Open Science Framework (ME89S) in accordance with the Preferred Reporting Items for Systematic Reviews and Meta-analyses (PRISMA) reporting guideline. The Third People's Hospital of Chengdu determined that this study was exempt from ethics approval and consent to participate because personal information was not involved.

### Design and Search Strategies

Web of Science, PubMed, and Retraction Watch Database were searched from their inception to July 17, 2021, to identify retracted publications on microRNA. Retraction, microRNA, withdrawal, and their synonyms were used as search terms. Detailed search strategies for each database are shown in the eMethods in Supplement 1. Additionally, PubPeer was referenced to examine the public response or comments on included retractions.

### Eligibility Criteria and Study Selection

Studies meeting the following criteria were included: they were about microRNA research; they were retracted articles regardless of reasons; and they were journal articles not conference abstracts. No restriction was imposed on language, participants, intervention, or comparison. Eligible articles were screened and selected by 2 investigators (H.Z. and Y.J.) according to these inclusion criteria independently. Disagreements between investigators were discussed with the third investigator (S-W.L.) until an agreement was reached and recorded with rationales.

### Data Extraction and Selection as Control Studies

**Figure 1.**
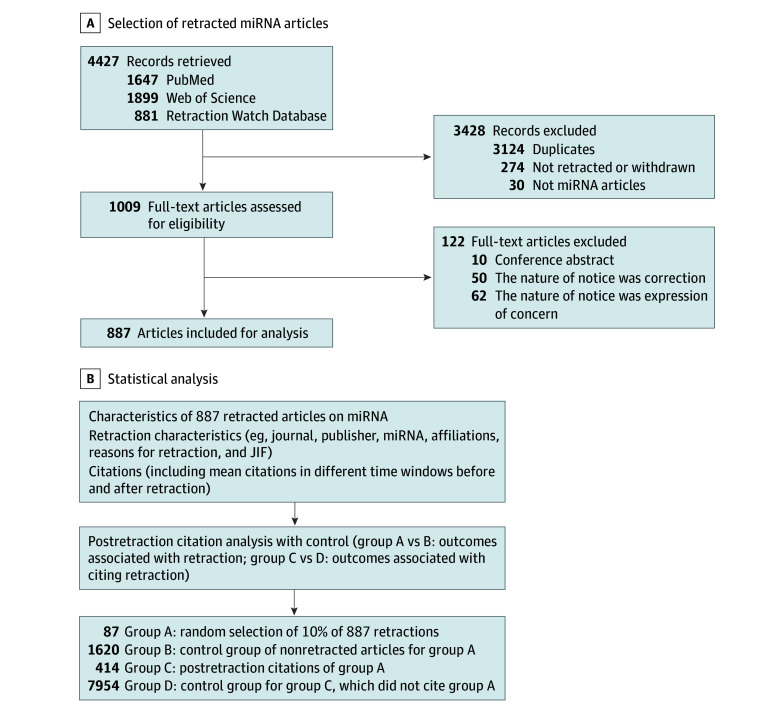
Study Flowchart JIF indicates journal impact factor; miRNA , microRNA.

The following characteristics of eligible articles were extracted: title, journal, publisher, specific microRNA, disease, affiliations, reasons for retraction, number of authors, dates of publication and retraction, nature of retraction notice, and country of authors. Retraction reasons were defined and categorized according to Retraction Watch Database User Guide Appendix B: Reasons.^16^ Additionally, Web of Science was used to retrieve data on citations of retracted articles, along with the journal impact factor (JIF) of the retracting journal for the calendar year before publication and for 2020 and its 2020 five-year JIF. To investigate the association of the retraction with further citations, a random selection of 10% of retracted articles (group A) based on an arbitrary seed number (set to 5 in this study) was performed (articles were ranked according to their publication date), and an analysis of their detailed citations was conducted. A set of nonretracted control articles (group B) from the same journals in the same year and month of publication and with the same number of authors was identified in PubMed. Studies citing retracted articles in group A were labeled as group C. The same method used to identify group B articles was used to find another set of control articles (group D) for group C (articles citing retractions); however, group D included articles regardless of whether they were retracted. The study design is shown in Figure 1.

### Statistical Analysis

Statistical analysis was performed with R statistical software version 4.2.2 (R Project for Statistical Computing).^17^ Continuous data following a normal distribution were presented as means with 95% CIs and compared using the Student *t* test. Continuous data that do not follow a normal distribution are expressed as median (IQR) and compared using the Mann-Whitney *U* test. Categorical data were reported as counts and percentages and compared using a χ^2^ test. All statistical tests were 2-sided, and values of *P* < .05 were considered statistically significant. Quality assessment was not conducted on retracted articles owing to the varieties of misconduct and removal of the full text by journals. Data were analyzed from September 2021 through March 2023.

## Results

### Retracted microRNA Studies and Their Characteristics

A total of 4427 records were identified from PubMed, Web of Science, and Retraction Watch Database. After removal of duplicates, 1303 records were available for screening of abstracts. The screening excluded 20 records that were not about microRNA and 274 records that were not retracted or withdrawn articles. Finally, 887 records met eligible criteria after we excluded 10 records of conference abstracts and 112 records of notices for correction or expression of concern (Figure 1). Of included records, 766 articles (86.36%) and 740 articles (83.43%) were labeled as retractions by Retraction Watch and PubPeer, respectively. There were 435 articles that received comments or attention in PubPeer (49.04%). Retracted articles were published between 1999 and 2021, with 36 articles not providing a retraction year. The first retraction of microRNA articles was in 2003 and initiated by *Nature*.^18^ As shown in eFigure 1 in Supplement 1, the number of retracted articles increased by year and articles published in 2019 had the most retractions. In accordance with preestablished criteria (eMethods in Supplement 1), 9574 control articles were identified, for a total of 10 461 articles.

The Table shows characteristics of retracted articles on microRNAs. Among all retracted articles, 842 articles (94.93%) had authors who were from a single country, 9 articles (1.01%) did not report author countries, and 36 articles (4.06%) had authors from multiple countries. China, the US, and Iran were the 3 most frequently reported countries among articles with authors from a single country, with 756 articles (89.79%; 85.23% of all articles), 39 articles (4.63%), and 20 articles (2.38%), respectively. Additionally, among 757 articles from hospitals, 731 articles (96.57%) came from China. Most retracted articles (782 articles [88.16%]) focused on a single microRNA, with miR-21, miR-124, and miR-155 the 3 most frequently reported microRNAs. Cancer research contributed more than two-thirds of retracted miRNA articles (617 articles [69.56%]); 799 retracted articles reported diseases, and 162 articles reported long noncoding RNA (lncRNA).

**Table.** Characteristics of Retracted Studies

Characteristic	Studies, No. (%)
Country	
Single	842 (94.93)
China	756 (89.79)
US	39 (4.63)
Iran	20 (2.38)
Other	27 (3.21)
Multiple	36 (4.06)
Not reported	9 (1.01)
Citation after retraction, No. (No. citations)	
Not available^a^	64
0 citations	202 (0)
1 to <50 citations	603 (4365)
50 to <100 citations	12 (936)
≥100 Citations	6 (1026)
microRNA	
Single	782 (88.16)
miR-21	25 (2.82)
miR-124	19 (2.14)
miR-155	14 (1.58)
Other	724 (81.62)
Multiple	105 (11.84)
2020 JIF, median (IQR)	4.06 (3.507-5.157)
Top 10 retraction reasons	
Paper mill^b^	290 (32.69)
Investigation by third party	267 (30.10)
Concerns or issues about data	235 (26.49)
Duplication of images	234 (26.38)
Unreliable results	210 (23.68)
Investigation by journal or publisher	160 (18.04
Withdrawal	136 (15.33)
Concerns or issues about images	128 (14.43)
Author unresponsive	117 (13.19)
Duplication of data	75 (8.46)
Disease	
Cancer	617 (69.56)
Lung cancer	81 (9.13)
Liver cancer	50 (5.64)
Gastric cancer	50 (5.64)
Osteosarcoma	48 (5.41)
Other cancer	436 (49.15)
Other disease	182 (20.52)
Not reported	88 (9.92)
No. of authors, median (IQR)	5 (4-7)
Months from publication to retraction, median (IQR)	22.03 (10.30-39.00)

Abbreviation: JIF, journal impact factor.

^a^ Citation was marked as not available if the study was not indexed by Web of Science.

^b^ Organizations selling fake work and authorships to researchers.

### Retraction Characteristics

Among retracted articles, the exact date of retraction was unknown for 98 articles (11.05%). For 789 articles with reported retraction dates, the median (IQR) time from publication to retraction was 22.03 (10.30-39.00) months. There were 847 articles retracted directly, while in 40 articles (4.51%), the journal published a correction or expression of concern before retraction. The time from notice of correction or concern to retraction varied, with a median (IQR) time of 8.9 (3.975-10.95) months. The shortest time from correction or expression of concern to retraction was less than 1 month, while the longest time was more than 6 years. Analysis of retraction reasons found that 870 articles (98.08%) were retracted because of 1 to 6 reasons, with a small proportion of articles retracted for more than 6 reasons. Data problems (including data in the form of images) and publication by paper mills were the main reasons that articles were retracted (Table; eTable 4 and eFigure 2 in Supplement 1).

**Figure 2.**
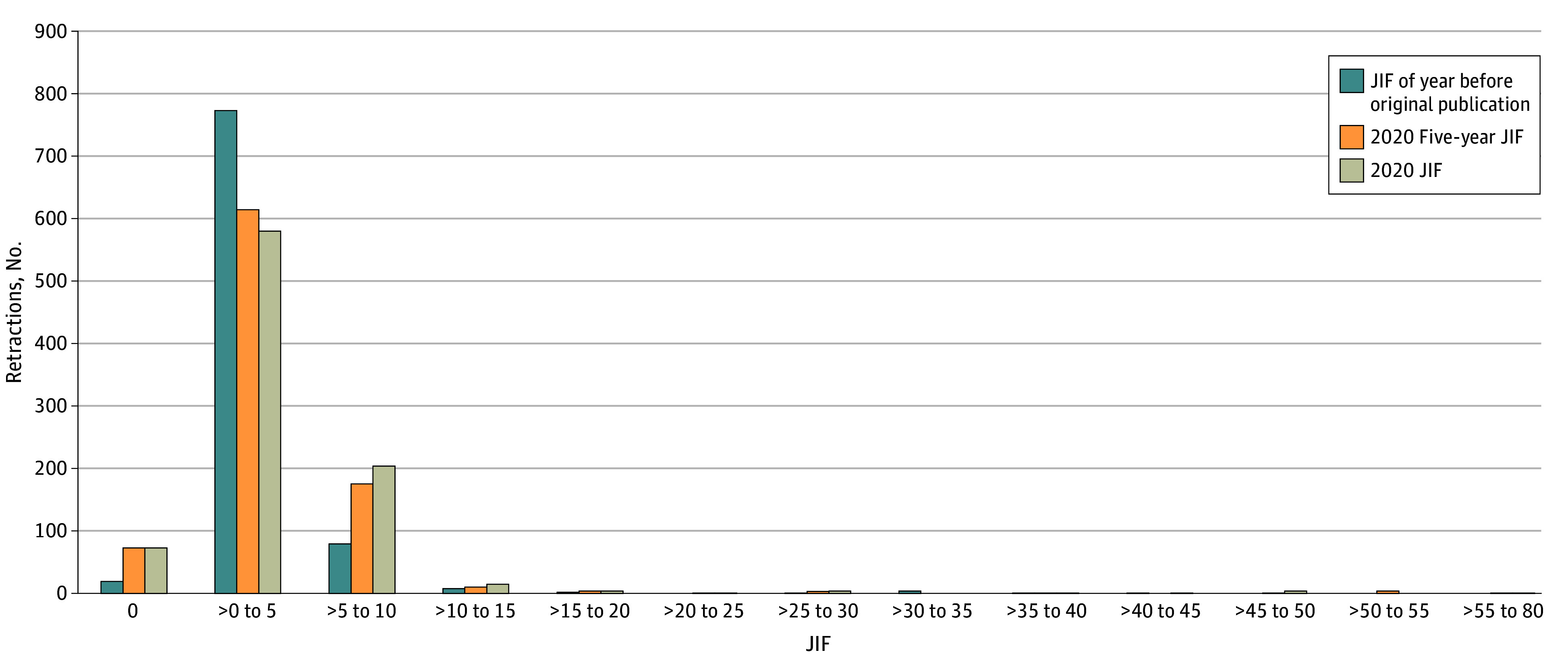
Numbers of Retractions by Journal Impact Factor (JIF) Journals marked zero on the x-axis had no JIF or were not indexed Science Citation Index or Science Citation Index Expanded journals.

Retractions of microRNA articles were made by 62 publishers and 204 journals. As shown in eTable 1 in Supplement 1, 16 publishers (25.81%) contributed 777 retractions (87.60%). One reason for this proportion was that the 16 publishers had more journals involved than the other 46 publishers. More importantly, the top 17 journals with the most retractions were published by these 16 publishers. As shown in eTable 1 in Supplement 1, the *European Review for Medical and Pharmacological Sciences* alone contributed 122 retractions, which was more than the 110 retractions by 46 publishers with 69 journals; *Journal of Cellular Biochemistry* was second, with 91 retractions. We investigated the number of retractions and JIF and found that retractions tended to occur in journals with a low or no impact factor (Figure 2).

### Citations

**Figure 3.**
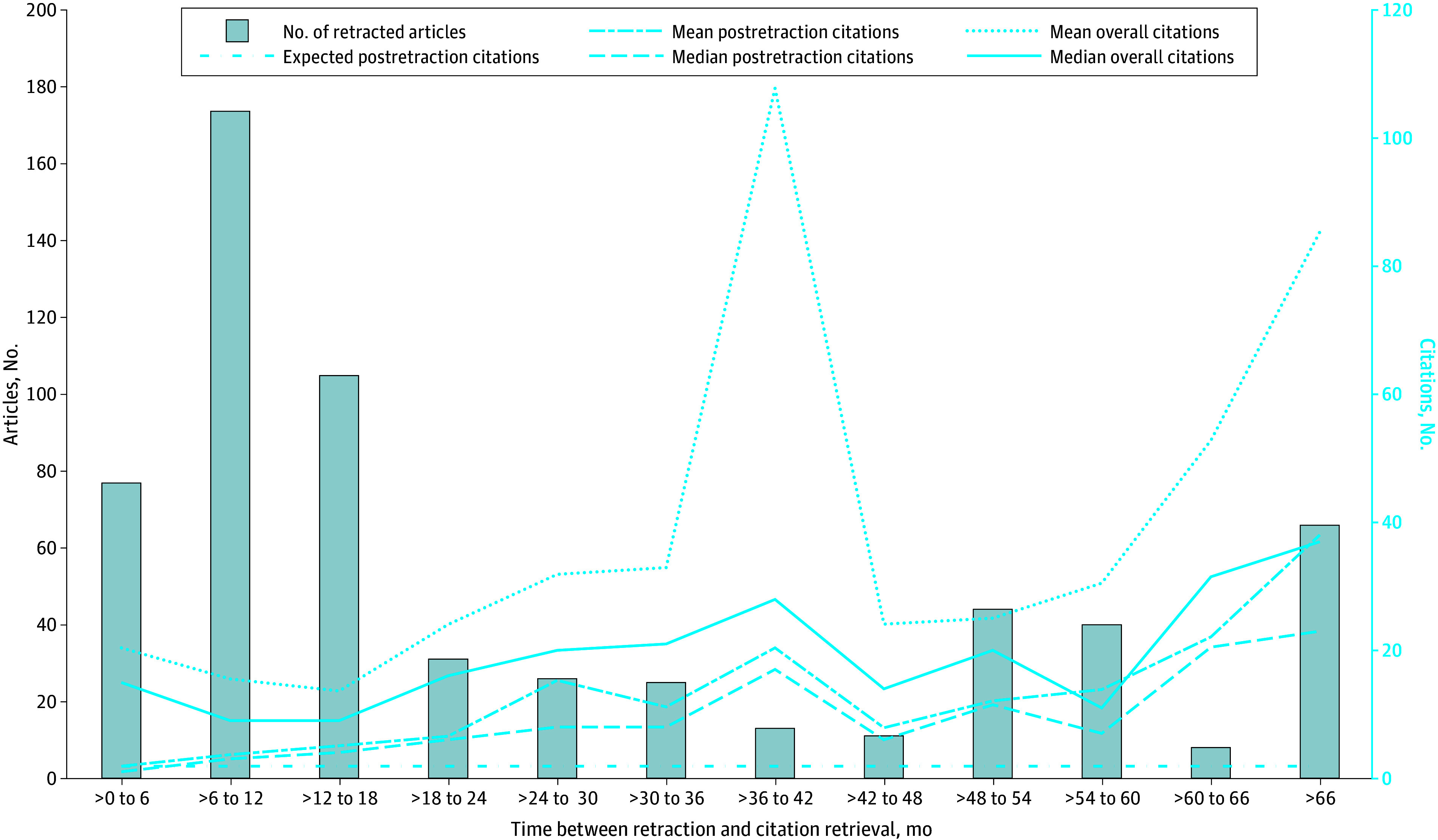
Mean and Median Citations by Time Window After Retraction The x-axis represents different time windows between retraction and citation retrieval (September 2021). The left y-axis shows the number of articles in each time window excluding those with zero citations between retraction and citation retrieval. Overall citations comprise citations before and after retraction. Expected postretraction citations were estimated from postretraction citations within 6 months of retraction.

Contrary to the expectation that retractions would be associated with reduced citations, retracted articles included in this systematic review were cited 6327 times after retraction (Table). Among 792 retracted articles with citations, 621 articles (78.41%) were cited at least once after retraction. Approximately 30% of retracted articles with citations (238 articles [30.05%]) were cited more after retraction than before retraction, including in top journals as ranked by *Journal Citation Reports* (eg, *Nature*),^19^ general Science Citation Index (SCI) and SCI-Expanded (SCIE) journals (eg, *Neuroscience Letters*), and journals that were not indexed by SCI or SCIE (eg, *Genetics and Molecular Research*). As shown in the Table, approximately 70% of retracted articles with citations had at least 1 citation after retraction (621 articles [70.01%]) and 6 articles had more than 100 citations after retraction. We examined the mean and median citations in different time windows after retraction and found that overall citations (comprising citations before and after retraction) and postretraction citations accumulated over time (eg, the median [IQR] number of postretraction citations was 1 [1-2] and 23 [9-44] citations at the first 6 and 66 months, respectively, between retraction and citation retrieval) (Figure 3), suggesting that retraction was not associated with reductions in citations of retracted articles.

**Figure 4.**
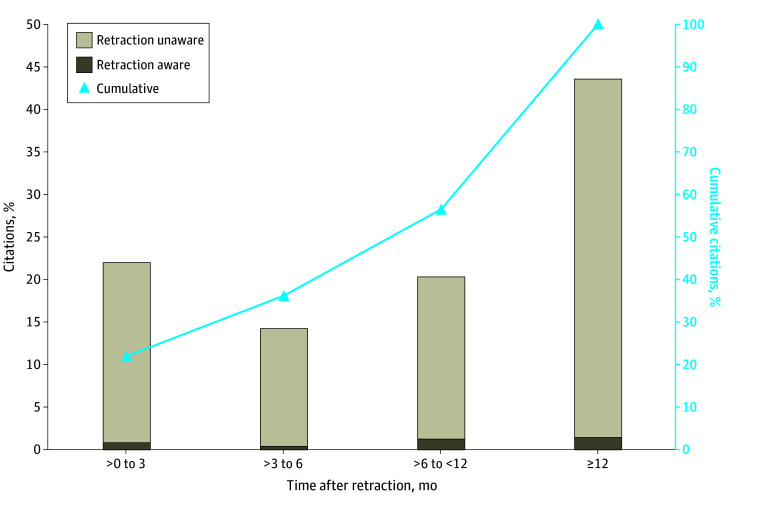
Percentages of Retraction-Unaware and Retraction-Aware Citations After Retractions

Citations after retraction were further analyzed in a random selection of retracted articles (89 articles [10.03%]) to investigate whether citing authors noted the status of publications (ie, the retracted state). There were 478 citations after the retraction of 87 articles (9.81%; excluding 2 articles without a date of retraction); 208 citations (43.51%) happened 12 months after retraction, and 19 citing articles (3.97%) noted retractions (Figure 4). Among all citations, 332 citations (69.46%) were in research articles, 141 citations (29.50%) were in reviews, and 2 citations (0.42%) were in proceedings of meetings; there was 1 citation (0.21%) each in a letter, editorial, and retraction notice. Among reviews, most citations were in the main text (136 citations [96.45%]), and among research articles, most citations were in the introduction (153 citations [46.08%]) and discussion (192 citations [57.83%]). However, 14 research articles (2.93%) cited the retraction in the methods section on data analysis or to establish models; 1 of these articles also incorporated the data in a new analysis. To investigate the association of retraction with citations, a total of 1620 nonretracted articles (group B) were identified in PubMed as controls for the 87 retracted articles (group A). No significant difference was found between retracted articles and their nonretracted control articles in overall citations or citations after retraction (eTable 2 in Supplement 1), but there were significant differences in citation difference (citations after retraction minus citations before retraction; mean rank, 689.26 vs 862.85; *P* = .001) (eTable 2 in Supplement 1) and odds of being cited more after retraction than before retraction (odds ratio, 0.62; 95% CI, 0.40-0.96) (eTable 3 in Supplement 1).

To test if articles citing retractions were more likely to be themselves retracted later, a set of 7954 articles (group D) that did not cite the 87 retracted articles were compared with 414 articles (group C) among 478 citing articles (7 citing articles were not found in PubMed, and no control articles were identified for 57 of 478 articles citing retractions). Of 414 articles citing retractions, 12 articles were retracted later compared with 36 of 7954 control articles retracted (odds ratio, 6.57; 95% CI, 3.39-12.72). After carefully checking, we found that 14 of 36 retracted articles in control articles cited other retracted articles but not the previously mentioned 87 retractions. These findings suggested that articles citing retractions were more likely to be retracted later.

## Discussion

### Principal Findings

This systematic review characterized citations of retracted articles and investigated the association of retractions with subsequent citations. A total of 887 microRNA articles retracted between 2003 and 2021 met inclusion criteria. More than 85% of retracted articles (756 articles [85.23%]) originated from China. Chinese authors of 731 retracted articles were affiliated with hospitals, consistent with previous reports^11,20^ that medical doctors were driven by substantial incentives, such as job promotion and cash rewards. Given that Chinese authorities stopped paying researchers cash rewards in 2020^21^ and stopped promoting or recruiting staff solely on the quantity of their publications, it seemed that Chinese authorities had been aware of the integrity issues of medical research in hospitals. In addition, the National Health Commission of China (in charge of hospitals) set up a special committee on June 3, 2021, to strengthen the integrity of medical research in hospitals.^22^ However, their new measures did not address the issue of postretraction citations.

Although some publishers have retracted a large number of articles from paper mills within a short time after publication, approximately 70% of included retracted microRNA articles were still cited by new articles after their retraction. Furthermore, approximately 30% of retracted articles (238 articles [30.05%]) were cited more frequently after retraction than before retraction. Unexpectedly, 43.51% of citations happened 12 months after retraction and 19 of 478 citing articles (3.97%) noted the retraction. The high rate of citation of articles after retraction was also found in a previous report^23^ and similar articles in other fields,^13,14^ which suggest that postretraction citation may be common and that not enough attention has been paid by some authors to retracted articles when citing articles. Overall citations (comprising citations before and after retraction) and postretraction citations accumulated over time. Furthermore, there were no significant differences between the sample of retracted articles and the control sample of nonretracted articles, regardless of overall citations and citations after retraction. In other words, retraction was not associated with changes in further citations, which echoes results of the study by Peng etal^24^ that retraction was not associated with a reduction in negative outcomes in science associated with problematic articles. The reason why retraction was not effective may be partly explained by the study by Xu et al,^25^ which compared official information channels (ie, releases of information through authorities, such as government agencies or organizations that have power in a particular area) with unofficial information channels (ie, contents posted are not released by the authorities) in disseminating retraction information from January 2005 to December 2014, finding that official channels were ineffective. Xu et al^25^ found that unofficial information channels provided additional retraction-related information (ie, retraction reasons and details of the retraction investigation processes) compared with official channels, which would be associated with reduced postretraction citations.^25^ The Committee on Publication Ethics (COPE) published the *COPE Retraction Guidelines* in November 2019, suggesting that official notices of retraction should state reasons for retraction.^1^ However, the inadequacy of official notices for retraction (eg, a lack of retraction reasons) was also found in biomedical literature retracted after 2019 (ie, in 2020 and 2021) in our study. Additionally, the Chinese Special Committee on Integrity of Medical Research government agency published the first batch of investigation and processing results involving 7 articles on June 8, 2021,^26^ and 3 articles had been retracted by journals at or before that time; 1 study was retracted 1 year later. However, any official notices (ie, expressions of concern or retraction) from journals about the other 3 articles were unavailable, while authors of these 3 articles had been accused falsification or fabrication of data, use of paper mills, and falsification or fabrication of data and images, respectively, and required retraction by the Chinese Special Committee on Integrity of Medical Research in 2021.

Retraction Watch and PubPeer are the main organizations for identifying questionable research. PubPeer is open to the public to leave comments about any articles. However, we found that only 435 of 887 articles (49.04%) received comments or attention, and these came from few individuals who were the most outspoken researchers. These findings suggest from another aspect that little attention was paid by the public or most researchers to journal retraction, which may have been associated with increased postretraction citations. Researchers are encouraged to check all references against Retraction Watch and PubPeer before submitting their manuscripts to journals. However, we found that approximately 85% of articles (766 articles [86.36%] in Retraction Watch and 740 articles [83.43%] in PubPeer) were labeled as retracted by these organizations. These findings suggest that verification against Retraction Watch and PubPeer may not be sufficient.

To keep retracted research out of scientific knowledge, we should pay attention not only to postretraction citations in individual articles, but also to the accuracy of existing knowledgebases, databases, and datasets. In this study, we found that some microRNA databases included data from retracted research on microRNAs. There are microRNA databases, including microRNA annotation, disease, target, cancer, and lncRNA databases, which are mainly curated by manual collection or by machine learning methods. Even if the curation was accurate, these databases did not have proper procedures to remove retracted microRNA information. Among 887 retracted microRNA articles, 799 articles reported diseases, 617 articles reported cancers, and 162 articles were about lncRNA, among other topics. We do not know if each previously mentioned database contained or failed to eliminate information from retracted articles, but we note that these databases contained information from retracted articles that were treated equally as data from unretracted articles (eg, 116 of 86 60 and 251 of 19 281 articles were retractions in miRTarBase^27^ and Human MicroRNA Disease Database,^28^ respectively, until early October 2021); thus thousands of articles may include retracted data (eg, TargetScan^29^ was cited 2085 times, miRTarBase^27^ was cited 309 times, miRDB^30^ was cited 420 times, and miRbase^31^ was cited 624 times according to a PubMed searching on September 22, 2021). We suggest that database maintainers and users should clean data periodically.

### Limitations

This study has several limitations. MicroRNA articles have recently been subject to numerous retractions and were used as an example to characterize postretraction citations; thus, the conclusion could vary over time as the list of retractions grows. Some detailed information, such as the date or reason of retraction, was unavailable despite attempts to contact journal offices, which may bias results. Additionally, key elements of official notice for retraction (elements should be stated according to COPE retraction guidelines) and their associated outcomes, as well as the degree of compliance with COPE retraction guidelines, remain unclear. An updated, comprehensive, and large-scale investigation study is warranted.

## Conclusions

This systematic review found that retraction was not associated with a reduction in citations of retracted articles. However, publications citing retracted articles as legitimate articles had a high risk of being retracted later. These findings suggest that researchers should verify the status from original sources before citing any references. Additionally, journals and publishers should implement stringent, preferably automated procedures to detect postretraction citations.

## Notes

Supplement 1. **eMethods.** Detailed Methods for This Systematic Review
eReferences.
**eTable 1.** Most Retraction Publishers and Journals **eTable 2.** Comparison of Citations Between Retracted Studies and Their Nonretracted Control Papers **eTable 3.** Odds Ratio of Citations Between Retracted Studies and Their Nonretracted Control Studies **eTable 4.** Analysis of Paper Mills **eFigure 1.** No. of Studies by Published Year or Retraction Year **eFigure 2.** Retraction Reasons

Supplement 2.
Data Sharing Statement
