# Supplementary material for: Citations of microRNA Biomarker Articles That Were Retracted: A Systematic Review
Source: JAMA Netw Open. 2024 Mar 21;7(3):e243173. doi: 10.1001/jamanetworkopen.2024.3173 (PMC10958238; doi:10.1001/jamanetworkopen.2024.3173)
Supplement: Supplement 1. — eMethods. Detailed Methods for This Systematic Review eReferences. eTable 1. Most Retraction Publishers and Journals eTable 2. Comparison of Citations Between Retracted Studies and Their Nonretracted Control Papers eTable 3. Odds Ratio of Citations Between Retracted Studies and Their Nonretracted Control Studies eTable 4. Analysis of Paper Mills eFigure 1. No. of Studies by Published Year or Retraction Year eFigure 2. Retraction Reasons [file jamanetwopen-e243173-s001.pdf]

Supplemental Online Content

Zhu H, Jia Y, Leung S. Citations of microRNA biomarker studies that were retracted: a systematic review. *JAMA Netw Open*. 2024;7(3):e243173.
doi:10.1001/jamanetworkopen.2024.3173

eMethods. Detailed Methods for This Systematic Review
eReferences.
eTable 1. Most Retraction Publishers and Journals
eTable 2. Comparison of Citations Between Retracted Studies and Their Nonretracted Control Papers
eTable 3. Odds Ratio of Citations Between Retracted Studies and Their Nonretracted Control Studies
eTable 4. Analysis of Paper Mills
eFigure 1. No. of Studies by Published Year or Retraction Year
eFigure 2. Retraction Reasons

This supplemental material has been provided by the authors to give readers additional information about their work.

© 2024 Zhu H et al. *JAMA Network Open*.

eMethods

Design

This systematic review aimed to characterize retractions on microRNA research and their post-retraction citation with additional control data, which was registered in Open Science Framework (OSF) with https://doi.org/10.17605/OSF.IO/ME89S in accordance with the Preferred Reporting Items for Systematic Reviews and Meta-Analyses (PRISMA) guideline[1]. PROSPERO was not adopted for protocol registration since the protocol did not meet the criteria of PROSPERO register, i.e., having no specific health-related outcome and quality assessment for included retractions.

Information sources and search strategies

PubMed as one of the most widely used life sciences and biomedical databases, is recommended as the references verified bibliographic source to minimize citation errors as well as an authoritative source of information about retractions for articles indexed in MEDLINE by ICMJE[2]. Web of Science is an important database platform for accessing global academic information, and indexes articles, reviews, letters, news and editorials from scholarly journals, books and proceedings in the sciences, engineering and technology, social sciences, arts and humanities, and other disciplines with citation alerts. Retraction Watch[3] is the main database tracking retractions on various subject groups including business/technology, basic life sciences, environment, health sciences, humanities, physical sciences and social sciences. Thus, searching Web of Science, PubMed and Retraction Watch Database from their inception to 17 July 2021 allowing a comprehensive identification of retractions on microRNA studies. Retraction, microRNA, withdrawal, and their synonyms were used as search terms. Detailed search strategies for each database are shown below:

For Web of Science, ((‘miR*’ OR ‘microRNA*’ OR ‘miRNA*’ in Title) AND (‘retract*’ OR ‘withdraw*’ in All Fields)) OR ((‘miR*’ OR ‘microRNA*’ OR ‘miRNA*’ in Abstract) AND (‘retract*’ OR ‘withdraw*’ in All Fields)).

For PubMed, (‘miR*’ OR ‘microRNA*’ OR ‘miRNA*’ in Title/Abstract) AND (‘retract*’ OR ‘withdraw*’ in All Fields).

Human microRNAs are usually expressed as ‘microRNA-*’ or ‘miR-*’ according to standard nomenclature. Those discovered before the standard nomenclature was established will retain their original names, such as let-7[4]. And, according to our previous search test, the use of ‘miR*’ OR ‘microRNA*’ is allowed to identify papers with title containing ‘let-7*’ when abstract searching is available. Thus, the Retraction Watch database was searched using the terms ‘miR*’ OR ‘microRNA*’ OR ‘let-7*’ in the title (abstract

© 2024 Zhu H et al. *JAMA Network Open*.

searching is not applicable for Retraction Watch) to filter out retracted studies on microRNAs. Additionally, PubPeer was checked to examine the public's response or comments on the included retractions.

Eligibility criteria and study selection

Studies meeting the following criteria were included: (a) they were about microRNA research; (b) they were retracted papers regardless of the retracted reasons; and (c) they were journal papers, not conference abstracts. No restriction was imposed on the language, participants, intervention, or comparison. Two investigators (HMZ and YLJ) screened and selected the eligible articles according to the above inclusion criteria independently. Disagreements between the two investigators were discussed and consulted with the third investigator (SWL) until an agreement was reached and recorded with rationales.

Data extraction and selection as control studies

The following characteristics of eligible studies were extracted and recorded: title, journal, publisher, microRNA, disease, affiliations, reasons for retraction, number of authors, dates of publication and retraction, nature of retraction notice, and country of authors. Retraction reasons were categorized according to Retraction Watch Database User Guide Appendix B: Reasons (https://retractionwatch.com/retraction-watch-database-user-guide/retraction-watch-database-user-guide-appendix-b-reasons/) which also provide the description/definition of reason. Additionally, Web of Science was used to retrieve data of citation of retracted papers (citation was marked as not available if the paper was not indexed by Web of Science), and journal impact factor (JIF) of the year before publication, 2020 JIF and 2020 five-year JIF of related journals. For evaluation of the retraction effects on further citations, a random selection of 10% of retracted papers (Group A) based on seed of 5 (papers were ranked according to their publication date before) and analysis of their detailed citation with non-retracted control papers were conducted. To ensure a fair comparison, we matched the retracted papers with the control papers that share same characteristics, including the date and journal of publication, and the number of authors. Matching of the date of publication would control temporal variations in public interest. Matching of journals would ensure comparison of papers within the same research scope, particularly subject areas, and same citation sources. The number of authors would be a control for relevant confounders such as scales of collaboration and cross-checking among authors. Retracted papers that had no match were excluded. Specifically, a set of non-retracted control papers (Group B) from the same journals in the same years and month of publication, and with the same number of authors were identified in PubMed and selected for retractions (Group A). Papers citing above retractions (Group A) were grouped as C. The method used to

© 2024 Zhu H et al. *JAMA Network Open*.

identify Group B was adopted to find another set of control papers (Group D, regardless of being retracted) for Group C (papers citing retractions).

Statistical analysis

Statistical analysis was performed on the above random selected papers (Group A) to study the effect of retraction on citation by comparing with a group of non-retracted control papers (Group B) identified in PubMed. Citing works (Group C) of Group A were compared with another set of control papers (Group D) identified by the same method to detect the risk of being retracted later. Statistical analysis was performed with R software (version 4.2.2)[5]. Continuous data following normal distribution were presented as mean with 95% confidence interval and compared using Student’s t-test. Continuous data that do not follow a normal distribution were expressed as median (interquartile range [IQR]) and compared using the Mann–Whitney U test. Categorical data were reported as counts and percentages and compared using a chi-square test. Comparison of continuous data among multiple groups was performed using one-way ANOVA or the Kruskal–Wallis test. All statistical tests were 2-sided, and values of $p < 0.05$ were considered statistically significant. Quality assessment was not conducted on retracted papers since the varieties of misconduct and removal of full text by the journals.

References

1. Page MJ, McKenzie JE, Bossuyt PM, et al. The PRISMA 2020 statement: an updated guideline for reporting systematic reviews. BMJ. Published online March 29, 2021:n71. doi:10.1136/bmj.n71

2. International Committee of Medical Journal Editors. Manuscript preparation: Preparing a manuscript for submission to a medical journal. Accessed July 19, 2023. https://www.icmje.org/recommendations/browse/manuscript-preparation/preparing-for-submission.html

3. Retraction Watch. The Retraction Watch Database. New York: The Center for Scientific Integrity. ISSN: 2692-465X. Published 2018. Accessed July 19, 2023. http://retractiondatabase.org/

4. Ambros V, Bartel B, Bartel DP, Burge CB et al. A uniform system for microRNA annotation. RNA. 2003;9(3):277–279. doi:10.1261/rna.2183803

5. The R Project for Statistical Computing. Accessed January 8, 2024. https://www.r-project.org/

© 2024 Zhu H et al. *JAMA Network Open*.

eTable 1. The most retraction publishers and journals

Publisher	Journal	No. of retractions	Total retractions	Average retractions
Wiley	Journal of Cellular Biochemistry	91	149	6.48
	Journal of Cellular Physiology	19		
	The rest 21 journals	39		
Verduci Editore	European Review for Medical and Pharmacological Sciences	122	122	122
Elsevier	Biochemical and Biophysical Research Communications	14	78	2.69
	The rest 28 journals	64		
Springer - Nature Publishing Group	Tumor Biology	37	67	5.58
	The rest 11 journals	30		
Spandidos	Oncology Reports	13	45	7.5
	Molecular Medicine Reports	11		
	The rest 4 journals	21		
Royal Society of Chemistry	RSC Advances	40	42	14
	The rest 2 journals	2		
Springer - Biomed Central (BMC)	The 17 journals	40	40	2.35
Portland Press	Bioscience Reports	30	32	16
	The rest 1 journal	2		
Taylor and Francis - Dove Press	OncoTargets and Therapy	20	32	10.67
	Cancer Management and Research	11		
	The rest 1 journal	1		
Springer	The 22 journals	31	31	1.41
Taylor and Francis	Artificial Cells, Nanomedicine, and Biotechnology	24	30	5
	The rest 5 journals	6		
PLoS	PLoS One	28	30	10
	The rest 2 journals	2		
e-Century Publishing Corporation	American Journal of Cancer Research	10	23	5.75
	The rest 3 journals	13		
Cognizant Communication Corporation	Oncology Research	23	23	23

© 2024 Zhu H et al. *JAMA Network Open*.

American Society for Biochemistry and Molecular Biology	The Journal of Biological Chemistry	22	22	22
Cell Physiol Biochem Press	Cellular Physiology and Biochemistry	11	11	11
The rest 46 publishers	The 69 journals	110	110	1.59

© 2024 Zhu H et al. *JAMA Network Open*.

eTable 2. The comparison of citations between retracted papers and their non-retracted control papers

Group	Sample size	Variable	Overall citations	Citations before retraction	Citations after retraction	Citation difference (after-before)
Retracted papers	87	Mean rank	896.75	982.69	811.56	689.26
Control papers	1620	Mean rank	851.70	847.09	856.28	862.85
Mann–Whitney U test		Z	-0.831	-2.520	-0.826	-3.202
		P value	0.406	0.012	0.409	0.001

© 2024 Zhu H et al. *JAMA Network Open*.

eTable 3. The odds ratio of citations between retracted papers and their non-retracted control papers

	Cited more after retraction than before retraction		Total	Chi-square test	
	Yes	No		OR (95% CI)	*p* value
Retracted papers	36	51	87	0.618	0.030
Control papers	864	756	1620	(0.399-0.957)	

© 2024 Zhu H et al. *JAMA Network Open*.

eTable 4. The analysis of paper mills

Retraction characteristics		Retraction counts
Retraction year	Unknown	2
	2017	2
	2018	0
	2019	2
	2020	92
	2021	192
Country	China	289
	Germany	1
	USA	1
	Unknown	1
Publisher	Wiley	99
	Royal Society of Chemistry (RSC)	41
	Verduci Editore	41
	Spandidos	26
	Taylor & Francis	21
	Portland Press	11
	Cognizant Communication Corporation	10
	Cell Physiol Biochem Press	9
	Elsevier	8
	e-Century Publishing Corporation	5
	Springer	5
	Taylor and Francis - Dove Press	4
	Associação Brasileira de Divulgação Científica (ABDC)	2
	IOS Press	2
	Springer - Nature Publishing Group	2
	Cellular Physiol Biochem Press	1
	Mary Ann Liebert	1
	Nature Publishing Group	1
	Springer - BioMed Central (BMC)	1

© 2024 Zhu H et al. *JAMA Network Open*.

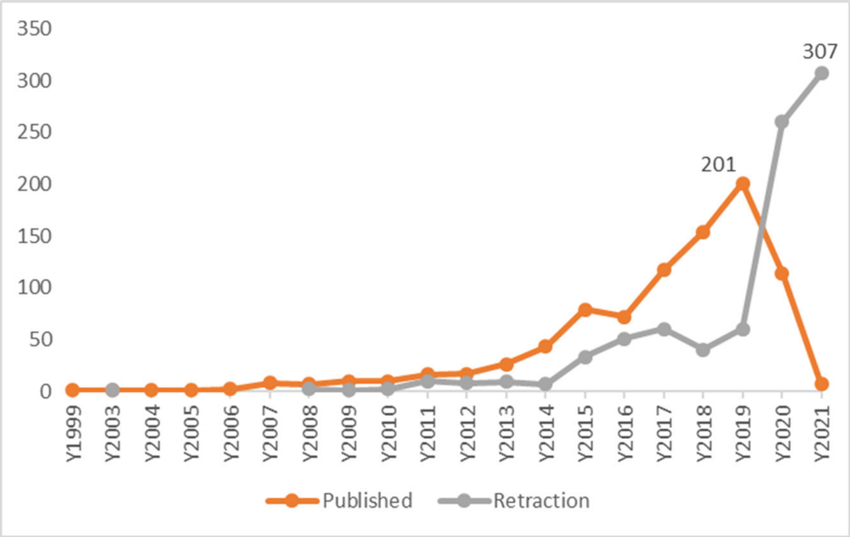

eFigure 1. The No. of papers varied according to published year or retraction year

© 2024 Zhu H et al. *JAMA Network Open*.

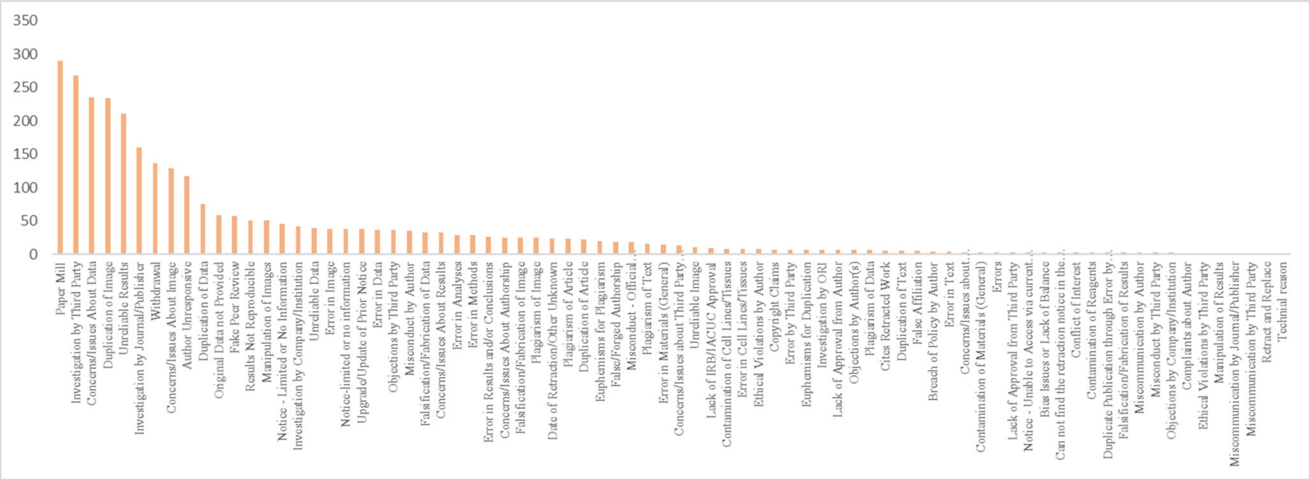

eFigure 2. Retraction reasons

© 2024 Zhu H et al. *JAMA Network Open*.
