# Supplementary material for: Citations of microRNA Biomarker Articles That Were Retracted: A Systematic Review
Source: JAMA Netw Open. 2024 Mar 21;7(3):e243173. doi: 10.1001/jamanetworkopen.2024.3173 (PMC10958238; doi:10.1001/jamanetworkopen.2024.3173)
Supplement: Supplement 2. — Data Sharing Statement [file jamanetwopen-e243173-s002.pdf]

Data Sharing Statement

Zhu. Citations of microRNA Biomarker Studies That Were Retracted: A Systematic Review. *JAMA Netw Open*. Published online March 21, 2024. doi:10.1001/jamanetworkopen.2024.3173

Data
Data available: Yes
Data types: Data (not involving human participants)
How to access data: The data will be provided as a supplement to the paper. **When available:** With publication

Supporting Documents
Document types: None

Additional Information
Who can access the data: The data will be provided as a supplement to the paper for anyone who has access to the paper.
Types of analyses: Basic statistics.
Mechanisms of data availability: Without investigator support.
